# Attitudes towards lung cancer screening in a population sample

**DOI:** 10.1111/hex.12819

**Published:** 2018-08-07

**Authors:** Stephanie E. Smits, Grace M. McCutchan, Jane A. Hanson, Kate E. Brain

**Affiliations:** ^1^ Division of Population Medicine Cardiff University Cardiff UK; ^2^ NHS Wales Health Collaborative Wales Cancer Network Cardiff UK

**Keywords:** attitudes, beliefs, cancer, lung cancer, quantitative, screening

## Abstract

**Background:**

Lung cancer is the leading cause of cancer death worldwide. Routine UK lung cancer screening is not yet available, thus understanding barriers to participation in lung screening could help maximize effectiveness if introduced.

**Methods:**

Population‐based survey of 1007 adults aged 16 and over in Wales using random quota sampling. Computer‐assisted face‐to‐face interviews included demographic variables (age, gender, smoking, social group), four lung cancer belief statements and three lung screening attitudinal items. Determinants of lung screening attitudes were examined using multivariable regression adjusted for age, gender, social group and previous exposure to lung campaign messages.

**Results:**

Avoidance of lung screening due to fear of what might be found was statistically significantly associated with negative lung cancer beliefs including fatalism (aOR = 8.8, 95% CI = 5.6‐13.9, *P* ≤ 0.001), low perceived value of symptomatic presentation (aOR = 2.4, 95% CI = 1.5‐3.9, *P* ≤ 0.001) and low treatment efficacy (aOR = 0.3, CI = 0.2‐0.7, *P* ≤ 0.01).

Low perceived effectiveness of lung screening was significantly associated with fatalism (aOR = 6.4, 95% CI = 3.5‐11.7, *P* ≤ 0.001), low perceived value of symptom presentation (aOR = 4.9, 95% CI = 2.7‐8.9, *P* ≤ 0.001) and low treatment efficacy (aOR = 0.1, 95% CI = 0.1‐0.3, *P* ≤ 0.001). In contrast, respondents who thought lung screening could reduce cancer deaths had positive beliefs about lung cancer (aOR = 0.4, 95% CI = 0.2‐0.7, *P* ≤ 0.001) and its treatment (aOR = 6.1, 95% CI = 3.0‐12.6, *P* ≤ 0.001).

**Conclusion:**

People with negative beliefs about lung cancer may be more likely to avoid lung screening. Alongside the introduction of effective early detection strategies, interventions are needed to modify public perceptions of lung cancer, particularly for fatalism.

## 1 INTRODUCTION

Lung cancer is the leading cause of cancer‐related death worldwide.^1^ Five‐year cancer survival rapidly decreases the later lung cancer is diagnosed, due to limited treatments options. In the UK, when lung cancer is diagnosed at the earliest stage (Stage I), 56% of patients can expect to survive for one year or more, in comparison with 14% of patients diagnosed at the most advanced stage (Stage IV).^2^ With 78% of UK non‐small‐cell lung cancer cases diagnosed in the later stages of disease (Stage III or IV),^2^ there is a need to explore strategies to diagnose lung cancer earlier.

Currently, diagnostic testing for suspected lung cancer in the UK requires symptomatic patients to present to a healthcare professional for referral for further investigation. This approach relies on the patient and healthcare professional accurately appraising symptoms, which may be problematic for early diagnosis of lung cancer.^3^ Lung cancer symptoms are hard to detect in the early stages, due to misattribution to smoking habit, comorbidities or other benign causes.^4^, ^5^

Evidence suggests that low‐dose computed tomography (CT) screening is effective in detecting early‐stage lung cancer.^6^ The US National Lung Screening Trial reported a 20% reduction in lung cancer mortality^6^ and is currently the standard of care in the United States.^7^ Although not routinely available in the UK, trials are ongoing across Europe to assess the effectiveness of CT lung screening among high‐risk groups.^8^, ^9^, ^10^, ^11^ In the event that lung screening for high‐risk populations is introduced routinely in the UK, it is important to understand the barriers to participation and develop interventions to encourage those who are eligible to engage in lung screening in order to optimize its impact and maintain cost‐effectiveness.

Previous studies of attitudes towards lung cancer screening suggest that smokers from socio‐economically deprived groups place lower value on the benefits of lung cancer screening, hold fatalistic beliefs about lung cancer as an untreatable disease or report stigma as a barrier to screening participation.^12^, ^13^ In addition, emotional barriers such as fear of lung cancer^14^ and the belief that the lungs are an untreatable organ^12^, ^15^ were reported to deter participation in lung screening trials. However, these studies have been restricted to samples of people over the age of 40.^12^, ^13^, ^15^, ^16^, ^17^, ^18^ Therefore, little is known about attitudes to lung screening in a population sample including younger age groups who may eventually become eligible for programmatic CT lung screening. Furthermore, there is limited evidence regarding the influences of general beliefs about lung cancer symptomatic presentation, survival and treatment on attitudes towards lung screening.

A population‐based survey was conducted to assess the influence of demographic variables, smoking status and beliefs about lung cancer and early symptomatic detection on lung cancer screening attitudes in a Welsh population sample. It was anticipated that current smokers, respondents from the lowest socio‐economic group and those with negative beliefs about lung cancer would have more negative attitudes towards lung cancer screening.

## 2 MATERIALS AND METHODS

### 2.1 Participants

Ethical approval was obtained to undertake a secondary analysis of population‐representative survey data gathered during February and March 2016, prior to the launch of the Welsh lung cancer awareness campaign in July 2016 (http://www.cancerresearchuk.org/health-professional/awareness-and-prevention/be-clear-on-cancer/lung-cancer-awareness-campaign-wales). Cancer Research UK commissioned a survey provider (Beaufort Research) to carry out a nationally representative survey of adults resident in Wales aged 16 years and over, as part of a commercial Omnibus survey to examine the impact of the campaign on lung symptom awareness.

Pre‐campaign survey data were collected from a total of 1007 adults. The number of people who declined to participate was not recorded, thus the characteristics of survey decliners are unknown.

### 2.2 Design/procedure

The survey used random quota sampling based on neighbourhoods classified according to census characteristics. The Omnibus sample is designed to be representative of the adult population resident in Wales aged 16 and over, with Lower Layer Super Output Area (LSOA) as the unit of sampling. Sixty‐nine interviewing points throughout Wales were selected with probability proportional to resident population, after stratification by local authority and social group based on occupation. Social group was recorded in four categories using the National Readership Survey grades, based on the occupation of the household's chief income earner: AB (higher and intermediate managerial, administrative and professional), C1 (supervisory, clerical and junior managerial, administrative and professional), C2 (skilled manual workers) and DE (semiskilled and unskilled manual workers, state pensioners, casual and lowest grade workers, and unemployed with state benefits only). Categories were combined to cluster participants by social group: ABC1 participants were considered high socio‐economic status, and the C2DE participants were considered low socio‐economic status.

Within each sampling point, quota sample controls of age and social group within gender were set for the selection of respondents. Quotas were set to reflect the individual demographic profile of each selected point. A fresh sample of interviewing locations and individuals was selected for each survey, and no more than one person per household was interviewed. Respondents completed a computer‐assisted interview in the presence of a trained interviewer. Data were weighted by age group within gender within local authority grouping, so that the sample profiles matched those of people aged 16 years and over in Wales derived from the 2011 Census.

### 2.3 Measures

Survey measures included demographic characteristics (age, gender, social group), smoking history (smoke up to 20 cigarettes a day, smoke 20 or more cigarettes a day, used to smoke, never smoked), beliefs about lung cancer and attitudes towards lung cancer screening. Prior exposure to lung campaign messages was measured by the following questions: “Have you seen, heard or read any adverts, publicity or other types of information in the last couple of months which focused on the subject of lung cancer?” Response options were “yes,” “no” and “don't know/can't remember.” A brief description of lung screening was given: *Now I'm going to read you some statements that are sometimes made about cancer screening (eg, a mammogram for breast cancer screening, a poo testing kit for bowel cancer screening). Thinking about lung screening (ie, a chest scan or X‐ray), can you tell me how much you agree or disagree with each of the following statements?* Items relating to lung cancer screening attitudes and beliefs about lung cancer were adapted from the ABC measure.^19^ Lung cancer beliefs were assessed with four items: “I would not want to know if I had lung cancer” reflecting cancer fatalism; “Going to my GP/doctor early with a symptom of lung cancer makes no difference to my chances of surviving lung cancer” reflecting perceived value of symptom presentation; “If lung cancer is diagnosed early, it is more likely to be treatable” reflecting beliefs about treatment; and “If I had a cough, I would be worried about wasting the GP/doctor's time” reflecting beliefs about symptomatic presentation. Attitudes towards lung cancer screening were assessed using three items: “I would be so worried about what might be found at lung cancer screening that I would prefer not to go”; “I don't think there is any point going for lung cancer screening because it won't affect the outcome”; and “Lung screening could reduce my chances of dying from cancer.” Response options were strongly agree, agree, disagree and strongly disagree. “Don't know” responses were recorded. Responses were recoded for analysis purposes, with strongly agree and agree combined to create “agree,” disagree and strongly disagree combined to create “disagree.” “Don't know” responses were counted as missing.

### 2.4 Statistical analysis

Descriptive statistics were used to summarize the demographic characteristics of the sample and to assess missing and “don't know” responses. Chi‐square univariable tests were used to examine the influence of smoking history, age, gender, social group and lung cancer beliefs on endorsement of attitudes to individual lung cancer screening items. Multivariable regression modelling was carried out to examine the influence of smoking history and lung cancer beliefs on lung cancer screening attitudes (individual items), adjusting for age, gender, social group combined and prior exposure to lung cancer messages. The significance level was set at *P* < 0.01 to adjust for multiple testing. To account for nonrepresentativeness, a weight was applied to the data based on age and gender within local authority in Wales.

## 3 RESULTS

Of a total of 1007 participants, 295 (29%) were aged 16‐34, 328 (33%) aged 33‐54 and 383 (38%) aged over 55 (see Table 1). There were 518 females (51%) and 489 males (49%), with 406 (41%) from the social group ABC1 and 596 (60%) from the social group C2DE. Most of the sample had never smoked (n = 433, 43%), 286 (28%) used to smoke, 259 (26%) currently smoked up to 20 cigarettes a day and 28 (3%) currently smoked more than 20 cigarettes a day (see Table 1). For univariable and multivariable analysis purposes, “smoke up to 20 a day” and “smoke over 20 a day” were combined to create a “currently smoke” category.

**Table 1:** Participant characteristics

Variable	Descriptive statistic n (%)	
	Unweighted	Weighted
Age		
16‐34	285 (28%)	295 (29%)
33‐54	282 (28%)	328 (33%)
55+	439 (44%)	383 (38%)
Gender		
Male	439 (44%)	489 (49%)
Female	568 (56%)	518 (51%)
Social group		
ABC1	412 (41%)	406 (40%)
C2DE	590 (59%)	596 (60%)
Smoking status		
Never smoked	445 (44%)	433 (43%)
Used to smoke	291 (29%)	286 (28%)
Smoke up to 20 a day	243 (24%)	259 (26%)
Smoke over 20 a day	27 (3%)	28 (3%)
Exposure to lung messages		
Yes	511 (51%)	515 (52%)
No	486 (49%)	483 (48%)
Lung cancer beliefs		
I would not want to know if I had lung cancer		
Agree	164 (17%)	168 (17%)
Disagree	802 (83%)	801 (83%)
Going to my GP/doctor early with a symptom of lung cancer makes no difference to my chances of surviving cancer		
Agree	172 (18%)	170 (18%)
Disagree	771 (82%)	777 (82%)
If lung cancer is diagnosed early, it is more likely to be treatable		
Agree	897 (94%)	905 (94%)
Disagree	60 (6%)	55 (6%)
If I had a cough, I would be worried about wasting the GP/doctor's time		
Agree	361 (37%)	358 (37%)
Disagree	609 (63%)	615 (63%)

### 3.1 Univariate analysis

#### 3.1.1 Avoidance of lung screening

**Figure 1:**
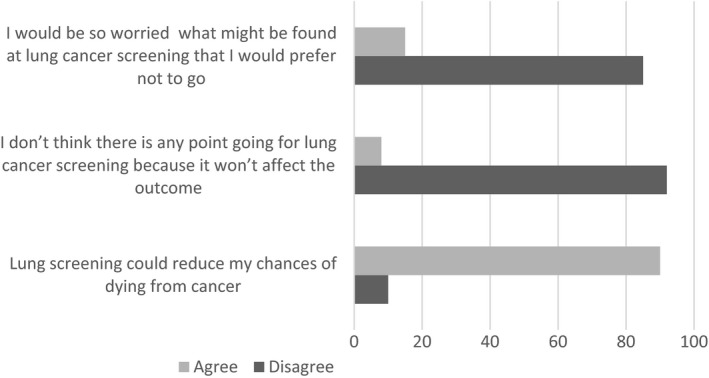
Summary of lung screening attitudes (data presented represents weighted data)

Fifteen per cent (n = 144) of the sample endorsed avoidance of lung screening due to fear of what might be found (Figure 1). Avoidance of lung screening was statistically significantly associated with fatalism (*P* ≤ 0.001), low perceived value of symptom presentation (*P* ≤ 0.001), having negative views about treatment (*P* ≤ 0.001) and worry about wasting the doctor's time (*P* ≤ 0.001) (see Table 2). Associations between lung screening attitudes and age, gender, social group, smoking status and exposure to lung messages were not statistically significant.

**Table 2:** Frequencies and univariate analysis for lung screening attitude “I would be so worried about what might be found at lung cancer screening that I would prefer not to go”

	Unweighted			Weighted		
	Agree (n = 141)	Disagree (n = 836)	Statistic	Agree (n = 144)	Disagree (n = 832)	Statistic
Age						
16‐34	43 (16%)	233 (84%)	Χ^2^ (_2_) = 0.78, *P* = 0.68	43 (15%)	242 (85%)	Χ^2^ (_2_) = 0.89, *P* = 0.64
33‐54	41 (15%)	232 (85%)		51 (16%)	268 (84%)	
55+	57 (13%)	370 (87%)		50 (13%)	321 (87%)	
Gender						
Male	65 (15%)	360 (85%)	Χ^2^ (_1_) = 0.34, *P* = 0.56	73 (15%)	400 (85%)	Χ^2^ (_1_) = 0.24, *P* = 0.62
Female	76 (14%)	476 (86%)		71 (14%)	432 (86%)	
Social group						
ABC1	51 (12%)	357 (88%)	Χ^2^ (_1_) = 1.98, *P* = 0.16	52 (13%)	350 (87%)	Χ^2^ (_1_) = 1.49, *P* = 0.22
C2DE	90 (16%)	475 (84%)		91 (16%)	479 (84%)	
Smoking status						
Never smoked	54 (12%)	383 (88%)	Χ^2^ (_2_) = 3.79, *P* = 0.15	55 (13%)	370 (87%)	Χ^2^ (_2_) = 2.22, *P* = 0.33
Used to smoke	40 (15%)	234 (85%)		41 (15%)	227 (85%)	
Currently smoke	47 (18%)	219 (82%)		48 (17%)	236 (83%)	
Exposure to lung messages						
Yes	73 (15%)	431 (85%)	Χ^2^ (_1_) = 0.00, *P* = 0.99	76 (15%)	432 (85%)	Χ^2^ (_1_) = 0.01, *P* = 0.94
No	67 (15%)	396 (85%)		67 (15%)	392 (85%)	
Lung cancer beliefs						
I would not want to know if I had lung cancer						
Agree	78 (49%)	82 (51%)	Χ^2^ (_1_) = 188.05, *P* = 0.000***	79 (48%)	85 (52%)	Χ^2^ (_1_) = 180.78, *P* = 0.000***
Disagree	56 (7%)	737 (93%)		58 (7%)	732 (93%)	
Going to my GP/doctor early with a symptom of lung cancer makes no difference to my chances of surviving cancer						
Agree	52 (31%)	115 (69%)	Χ^2^ (_1_) = 46.97, *P* = 0.000***	51 (31%)	114 (69%)	Χ^2^ (_1_) = 41.85, *P* = 0.000***
Disagree	79 (10%)	682 (90%)		84 (11%)	681 (89%)	
If lung cancer is diagnosed early, it is more likely to be treatable						
Agree	109 (12%)	772 (88%)	Χ^2^ (_1_) = 16.84, *P* = 0.000***	114 (13%)	774 (87%)	Χ^2^ (_1_) = 18.39, *P* = 0.000***
Disagree	19 (32%)	40 (68%)		19 (35%)	36 (66%)	
If I had a cough, I would be worried about wasting the GP/doctor's time						
Agree	74 (21%)	283 (79%)	Χ^2^ (_1_) = 18.08, *P* = 0.000***	73 (21%)	281 (79%)	Χ^2^ (_1_) = 14.77, *P* = 0.000***
Disagree	63 (11%)	535 (89%)		68 (11%)	535 (89%)	

***p≤0.001

#### 3.1.2 Low perceived effectiveness of lung screening

A total of 8% (n = 78) endorsed low perceived effectiveness of lung screening (see Figure 1). Low perceived effectiveness of lung screening was statistically significantly associated with fatalism (*P* ≤ 0.001), low perceived value of symptom presentation (*P* ≤ 0.001), having negative views about treatment (*P* ≤ 0.001) and worry about wasting the doctor's time (*P* ≤ 0.001) (see Table 3). Effects of age, gender, smoking status, social group and exposure to lung messages were not statistically significant.

**Table 3:** Frequencies and univariate analysis for lung screening attitude “I don't think there is any point going for lung cancer screening because it won't affect the outcome”

	Unweighted			Weighted		
	Agree (n = 83)	Disagree (n = 876)	Statistic	Agree (n = 78)	Disagree (n = 882)	Statistic
Age						
16‐34	20 (7%)	251 (93%)	Χ^2^ (_2_) = 0.84, *P* = 0.66	20 (7%)	262 (93%)	Χ^2^ (_2_) = 0.66, *P* = 0.72
33‐54	24 (9%)	247 (91%)		26 (8%)	290 (92%)	
55+	39 (9%)	377 (91%)		32 (9%)	330 (91%)	
Gender						
Male	38 (9%)	374 (91%)	Χ^2^ (_1_) = 0.18, *P* = 0.67	39 (9%)	422 (91%)	Χ^2^ (_1_) = 0.61, *P* = 0.81
Female	45 (8%)	502 (92%)		39 (8%)	460 (92%)	
Social group						
ABC1	26 (6%)	374 (94%)	Χ^2^ (_1_) = 3.70, *P* = 0.05	25 (6%)	370 (94%)	Χ^2^ (_1_) = 2.61, *P* = 0.11
C2DE	57 (10%)	498 (90%)		53 (9%)	508 (91%)	
Smoking status						
Never smoked	28 (7%)	402 (93%)	Χ^2^ (_2_) = 7.94, *P* = 0.02*	25 (6%)	394 (94%)	Χ^2^ (_2_) = 6.96, *P* = 0.03*
Used to smoke	22 (8%)	247 (92%)		21 (8%)	244 (92%)	
Currently smoke	33 (13%)	227 (87%)		32 (12%)	245 (88%)	
Exposure to lung messages						
Yes	42 (9%)	454 (91%)	Χ^2^ (_1_) = 0.04, *P* = 0.85	39 (8%)	462 (92%)	Χ^2^ (_1_) = 0.13, *P* = 0.71
No	41 (9%)	413 (91%)		39 (9%)	412 (91%)	
Lung cancer beliefs						
I would not want to know if I had lung cancer						
Agree	47 (30%)	112 (70%)	Χ^2^ (_1_) = 105.84, *P* = 0.000***	45 (27%)	119 (73%)	Χ^2^ (_1_) = 97.64, *P* = 0.000***
Disagree	33 (4%)	749 (96%)		31 (4%)	749 (96%)	
Going to my GP/doctor early with a symptom of lung cancer makes no difference to my chances of surviving cancer						
Agree	40 (24%)	123 (76%)	Χ^2^ (_1_) =70.07, *P* = 0.000***	37 (23%)	124 (77%)	Χ^2^ (_1_) = 64.98, *P* = 0.000***
Disagree	34 (4%)	722 (96%)		32 (4%)	729 (96%)	
If lung cancer is diagnosed early, it is more likely to be treatable						
Agree	56 (6%)	817 (94%)	Χ^2^ (_1_) = 47.46, *P* = 0.000***	55 (6%)	826 (94%)	Χ^2^ (_1_) = 42.12, *P* = 0.000***
Disagree	19 (33%)	39 (67%)		17 (32%)	37 (68%)	
If I had a cough, I would be worried about wasting the GP/doctor's time						
Agree	45 (13%)	306 (87%)	Χ^2^ (_1_) = 12.67, *P* = 0.000***	43 (12%)	305 (88%)	Χ^2^ (_1_) = 13.85, *P* = 0.000***
Disagree	35 (6%)	557 (94%)		32 (5%)	566 (95%)	

*p≤0.05 , ***p≤0.001

#### 3.1.3 Lung screening to reduce mortality

Ninety per cent (n = 859) of the sample agreed that lung screening could reduce chances of lung cancer death (Figure 1). Agreeing that lung screening could reduce chances of dying from cancer was associated with positive lung cancer beliefs reflecting lack of fatalism (*P* ≤ 0.001) and having positive views about treatment (*P* ≤ 0.001) (see Table 4). There were no statistically significant effects of any demographic variables.

**Table 4:** Frequencies and univariate analysis for lung screening attitude “Lung screening could reduce my chances of dying from cancer”

	Unweighted			Weighted		
	Agree (n = 857)	Disagree (n = 91)	Statistic	Agree (n = 859)	Disagree (n = 92)	Statistic
Age						
16‐34	247 (92%)	22 (8%)	Χ^2^ (_2_) = 3.39, *P* = 0.18	258 (92%)	22 (8%)	Χ^2^ (_2_) = 3.60, *P* = 0.17
33‐54	233 (88%)	33 (12%)		273 (88%)	38 (12%)	
55+	376 (91%)	36 (9%)		327 (91%)	32 (9%)	
Gender						
Male	381 (92%)	32 (8%)	Χ^2^ (_1_) = 2.52, *P* = 0.11	424 (92%)	38 (8%)	Χ^2^ (_1_) = 1.85, *P* = 0.17
Female	476 (89%)	59 (11%)		435 (89%)	54 (11%)	
Social group						
ABC1	366 (92%)	32 (8%)	Χ^2^ (_1_) = 1.49, *P* = 0.22	359 (92%)	33 (8%)	Χ^2^ (_1_) = 0.89, *P* = 0.35
C2DE	488 (89%)	58 (11%)		496 (89%)	58 (11%)	
Smoking status						
Never smoked	386 (91%)	39 (9%)	Χ^2^ (_2_) = 4.29, *P* = 0.12	374 (90%)	40 (10%)	Χ^2^ (_2_) = 3.32, *P* = 0.19
Used to smoke	250 (93%)	20 (7%)		246 (93%)	20 (7%)	
Currently smoke	221 (87%)	32 (13%)		238 (88%)	33 (12%)	
Exposure to lung messages						
Yes	438 (90%)	51 (10%)	Χ^2^ (_1_) = 0.47, *P* = 0.49	445 (90%)	50 (10%)	Χ^2^ (_1_) = 0.07, *P* = 0.79
No	410 (91%)	40 (9%)		406 (91%)	42 (9%)	
Lung cancer beliefs						
I would not want to know if I had lung cancer						
Agree	125 (81%)	29 (19%)	Χ^2^ (_1_) = 15.80, *P* = 0.000***	128 (81%)	30 (19%)	Χ^2^ (_1_) = 16.60, *P* = 0.000***
Disagree	712 (92%)	62 (8%)		712 (92%)	62 (8%)	
Going to my GP/doctor early with a symptom of lung cancer makes no difference to my chances of surviving cancer						
Agree	135 (87%)	21 (13%)	Χ^2^ (_1_) = 2.92, *P* = 0.09	133 (86%)	22 (14%)	Χ^2^ (_1_) = 3.80, *P* = 0.051
Disagree	685 (91%)	65 (9%)		690 (91%)	66 (9%)	
If lung cancer is diagnosed early, it is more likely to be treatable						
Agree	804 (92%)	67 (8%)	Χ^2^ (_1_) = 42.37, *P* = 0.000***	809 (92%)	70 (8%)	Χ^2^ (_1_) = 34.78 *P* = 0.000***
Disagree	35 (65%)	19 (35%)		33 (66%)	17 (34%)	
If I had a cough, I would be worried about wasting the GP/doctor's time						
Agree	306 (89%)	38 (11%)	Χ^2^ (_1_) = 0.92, *P* = 0.34	304 (89%)	39 (11%)	Χ^2^ (_1_) = 1.17, *P* = 0.28
Disagree	533 (91%)	52 (9%)		539 (91%)	53 (9%)	

***p≤0.001

#### 3.1.4 Logistic regression

Regression analysis was completed using the weighted data, adjusting for age, gender, social group and previous exposure to lung messages.

#### 3.1.5 Avoidance of lung screening

Negative lung cancer beliefs including fatalism (aOR = 8.8 CI = 5.6‐13.9, *P* ≤ 0.001), low perceived value of symptom presentation (aOR = 2.4, CI = 1.5‐3.9, *P* ≤ 0.001) and having negative views about treatment (aOR = 0.3, CI = 0.2‐0.7, *P* ≤ 0.01) showed a statistically significant association with not wanting to have lung screening due to being worried about what might be found (see Table 5). Smoking status and being worried about wasting the doctor's time were not statistically significantly associated with lung cancer screening avoidance due to worry about the outcome.

**Table 5:** Multivariable logistic regression models predicting agreement with lung cancer attitudes^a^, ^b^

	Q1. I would be so worried about what might be found at lung screening that I would prefer not to go			Q2. I don't think there is any point going for lung cancer screening because it won't affect the outcome			Q3. Lung screening could reduce my chances of dying from cancer		
	B (SE)	OR (95% CI)	*P*	B (SE)	OR (95% CI)	*P*	B (SE)	OR (95% CI)	*P*
Smoking status	0.17 (0.30)	1.19 (0.66‐2.13)	0.56	−0.18 (0.39)	0.84 (0.39‐1.79)	0.64	0.47 (0.35)	1.61 (0.81‐3.17)	0.17
I would not want to know if I had cancer	2.18 (0.23)	8.80 (5.58‐13.87)	0.000***	1.85 (0.31)	6.38 (3.49‐11.66)	0.000***	−0.91 (0.28)	0.40 (0.23‐0.70)	0.001***
Going to my GP/doctor early with a symptom of lung cancer makes no difference to my chances of surviving cancer	0.86 (0.25)	2.37 (1.45‐3.86)	0.001***	1.58 (0.31)	4.85 (2.65‐8.88)	0.000***	−0.17 (0.30)	0.84 (0.47‐1.53)	0.58
If lung cancer is diagnosed early, it is more likely to be treatable	−1.08 (0.40)	0.34 (0.16‐0.74)	0.007**	−2.01 (0.43)	0.13 (0.06‐0.31)	0.000***	1.81 (0.37)	6.12 (2.98‐12.56)	0.000***
If I had a cough, I would be worried about wasting the GP/doctor's time	0.47 (0.22)	1.60 (1.03‐2.49)	0.04*	0.71 (0.31)	2.04 (1.12‐3.72)	0.02*	0.14 (0.26)	1.15 (0.69‐1.90)	0.60

OR, odds ratio; CI, confidence interval

^*^ *P* ≤ 0.05

^**^ *P* ≤ 0.01

^***^ *P* ≤ 0.001

^a^Weighting for nonrepresentativeness in age and gender within local authority in Wales

^b^Adjusting for age, gender, social group and previous exposure to lung messages

#### 3.1.6 Low perceived effectiveness of lung screening

Negative lung cancer beliefs including fatalism (aOR = 6.4 CI = 3.5‐11.7, *P* ≤ 0.001), low perceived value of symptom presentation (aOR = 4.9, CI = 2.7‐8.9, *P* ≤ 0.001) and having negative views about treatment (aOR = 0.1, CI = 0.1‐0.3, *P* ≤ 0.001) showed a statistically significant association with low perceived effectiveness of lung screening (see Table 5). Smoking status and being worried about wasting the doctor's time were not statistically significantly associated with attitudes towards the efficacy of screening.

#### 3.1.7 Lung screening to reduce mortality

Positive lung cancer beliefs reflecting lack of fatalism (aOR = 0.4 CI = 0.2‐0.7, *P* ≤ 0.001) and positive views about treatment (aOR = 6.1, 95% CI = 3.0‐12.6, *P* ≤ 0.001) showed a statistically significant association with agreeing that lung screening could reduce chances of dying from cancer (see Table 5). Smoking status, beliefs about early presentation and being worried about wasting the doctor's time were not significantly associated with perceptions that lung screening could reduce lung cancer mortality.

## 4 DISCUSSION

To our knowledge, the present study was the first to test associations with lung cancer screening attitudes using quantitative survey methods in a population sample of adults over the age of 16. Attitudes towards lung cancer screening were generally positive, with over 90% of survey respondents believing that there was benefit to lung cancer screening in terms of lung cancer outcomes and survival, and may encourage participation in lung cancer screening. However, those who endorse negative beliefs about lung cancer may be more likely to avoid lung screening. Respondents who endorsed negative beliefs about lung cancer—reflecting fatalism, low perceived effectiveness of symptom presentation and negative views about treatment—were more likely to hold negative attitudes towards lung cancer screening. Smoking status was not significantly associated with attitudes towards lung cancer screening in the current study.

Our findings mirror those of previous studies that have examined participation in a colorectal cancer screening context, where over 90% of respondents in an Australian population‐based study held positive beliefs about colorectal cancer screening.^20^ In addition, positive beliefs about the benefits of colorectal cancer screening have been associated with increased anticipated uptake of screening^21^ and participation in screening.^20^, ^22^

The current study suggests that negative beliefs about lung cancer were associated with lung screening avoidance, particularly fatalism, suggesting that those who decline screening would prefer not to know if they have lung cancer, potentially due to fear of treatment and lung cancer death. Our findings are in line with previous research highlighting fear of lung cancer^12^, ^13^, ^14^ and fatalism, including beliefs about the treatment for lung cancer^12^, ^15^ as barriers to participation in lung cancer screening. It is likely that avoidance of lung screening and negative beliefs may reflect lung cancer stigma,^23^ possibly due to the relationship between lung cancer and smoking, and poor lung cancer outcomes.

The absence of an observed association between smoking status and lung cancer screening attitudes in our study contradicts the findings of previous studies, which have highlighted more negative screening attitudes among current smokers.^12^, ^24^ In addition, former smokers have been shown to be over‐represented in lung cancer screening trials.^25^, ^26^ Our contradictory findings are likely to reflect the limited representation of heavy smokers in our sample and consequent low statistical power. Poor representation from current smokers should be noted as a limitation of this study. Future work focusing on attitudes in heavy, moderate and light smokers would help to further understanding of the influence of nicotine dependence on screening attitudes. It should also be noted that the associations between lung cancer beliefs and lung screening attitudes may partly reflect shared method variance, where associations between variables can be inflated when measures are taken at the same point in time. Prospective longitudinal research should therefore be undertaken to examine the predictors of lung screening uptake and outcomes. Finally, we used an adapted version of the ABC measure in the absence of a validated measure of lung screening at the time of survey development. Future studies could consider using a recently developed and psychometrically validated measure of lung screening health beliefs.^27^

Our findings suggest that negative beliefs about lung cancer may deter participation in lung cancer screening. Therefore, addressing population beliefs about lung cancer is an important step before the implementation of a lung screening programme. Public awareness campaigns should focus on the benefits of lung cancer screening, where early detection increases survival through access to more effective treatments, to modify fatalistic beliefs about lung cancer survival and treatment.

## CONFLICT OF INTEREST

No conflict of interests to declare
